# Prevalence and Correlates of Discomfort and Acceptability of Acupuncture among Outpatients in Chinese Acupuncture and Moxibustion Departments: A Cross-Sectional Study

**DOI:** 10.1155/2013/715480

**Published:** 2013-06-24

**Authors:** Baoyan Liu, Huanfang Xu, Shengnan Guo, Jiani Wu, Jia Liu, Min Yee Lim, Zhishun Liu

**Affiliations:** ^1^Guang'anmen Hospital, China Academy of Chinese Medical Sciences, Beijing 100053, China; ^2^Beijing University of Chinese Medicine, Beijing 100029, China; ^3^China & Nanyang Technological University, 50 Nanyang Avenue, Singapore 639798

## Abstract

*Objective.* This study aims to give a profile of discomfort and acceptability of acupuncture, including the prevalence and association with demographic and acupuncture-related factors. *Methods.* A cross-sectional study was conducted in Beijing, China. Outpatients of acupuncture and moxibustion departments were recruited using purposive sampling. 925 subjects were interviewed with an anonymous questionnaire. Multinomial and binary logistic regression were used to analyze factors affecting discomfort and acceptability of acupuncture. *Results.* The average VAS value of 925 subjects' acupuncture discomfort was 2.66 ± 2.02, within the range of mild discomfort. Acupuncture was easily accepted by 81.1% of the subjects. Results of logistic regression were as follows: (1) subjects with a better knowledge of acupuncture, or a greater fear of pain or needles, experienced more “moderate to severe discomfort” and showed a decreased acupuncture acceptance (*P* < 0.001 or *P* < 0.01); (2) Acupuncture with less discomfort or implemented by a more qualified doctor was easy to be accepted (*P* < 0.001); (3) subjects aged 20–29 preferred to report “moderate to severe discomfort” while those aged 40–59 preferred to report “slight discomfort” (*P* < 0.001). *Conclusion.* Acupuncture is an acceptable therapy with less discomfort, which can be greatly affected by fear of pain or needles, age, knowledge of acupuncture, and professional title of acupuncturist.

## 1. Background

Acupuncture, as a main component of traditional Chinese medicine (TCM), has been adopted on diseases prevention and treatment for over 2,000 years in China. It exerts its effects through stimulating of acupoints with acupuncture needles, and thus triggers the body's own ability to prevent diseases [1]. Due to its excellent efficacy, acupuncture has been increasingly accepted and used by practitioners and patients worldwidely. 

A fact is that acupuncture is a minimally invasive therapy, which may induce anxiety and fear to some patients [2]. When acupuncturists insert needles or perform acupuncture manipulation, patients may produce various sensations, such as sourness, numbness, distending, or pain [3]. According to TCM theory, sensations induced by acupuncture are closely related to deqi, a traditional acupuncture terminology which describes the connection between acupuncture needles and the energy pathways of the body and is essential for curative effect [4]. In our opinion, acupuncture sensations are in essence negative emotional experience for patient whether belonging to deqi or not. From this perspective, we named them discomfort of acupuncture. This study is designed to answer these two questions. To what degree do patients experience discomfort of acupuncture? What are the influence factors? By finding answers for these two questions, acupuncturists can change the acupuncture regimen accordingly, and thus improve patients' experience of acupuncture.

Though acupuncture is more and more used all over the world, there are few profiles on its discomfort, acceptability and influence factors. Discomfort of acupuncture strongly affects its acceptability. Our study showed that 44.2% (69/156) of the outpatients were reluctant to choose acupuncture because of fear of needles. In addition, acupuncture acceptability could also be affected by patients' characterizations, demographic data, and disease pattern: predominance, with physical symptoms such as diseases of the musculoskeletal system or injury, was the striking characteristic among acupuncture patients [5, 6].

In short, profiles of acupuncture discomfort and acceptability are still uncertain. Moreover, personalized treatment is one of the acupuncture characteristics, and different conditions of patients directly influence the treatment protocol. Therefore, it is useful to understand factors associated with discomfort and acceptability of acupuncture. This pilot survey focuses on the discomfort and acceptability of acupuncture, and the associated demographic and acupuncture-related factors, to provide useful suggestions on relieving discomfort of acupuncture and improving acceptance and personalized acupuncture treatment. 

## 2. Methods

### 2.1. Study Design

A cross-sectional survey was conducted between May and August 2010 to assess the prevalence of discomfort and acceptability of acupuncture and associated factors. The study was conducted with a purposive sampling in three hospitals in Beijing: Guang'anmen Hospital affiliated to China Academy of Chinese Medical Sciences, Dongzhimen Hospital affiliated to Beijing University of Chinese Medicine, and Dongfang Hospital affiliated to Beijing University of Chinese Medicine, which were selected from a total of six top traditional Chinese medicine hospitals firstly, and then every two acupuncture consulting rooms were randomly selected from each of the three hospitals.

Outpatients from the six consulting rooms at the three selected hospitals, who were willing to be interviewed and were able to complete the questionnaire, participated in the survey. The subjects were asked to do the survey within 10 minutes after completing acupuncture treatment. As we did not collect any personal identifiable information, voluntary provision of information was deemed to be consent. Subjects were invited to complete an anonymous questionnaire (for more details see Supplementary Material available online at http://dx.doi.org/10.1155/2013/715480) under the guidance of trained interviewers. A total of 928 subjects were recruited from May 4th to August 31st. Data from 925 subjects were included in the analysis. Three incomplete questionnaires were not included.

### 2.2. Data Collection

#### 2.2.1. Information Collected

We collected data on subjects' discomfort of acupuncture (one item), acceptability of acupuncture (one item), demographic profile (three items), and acupuncture-related information (five items).

#### 2.2.2. Discomfort of Acupuncture

**Figure 1:**
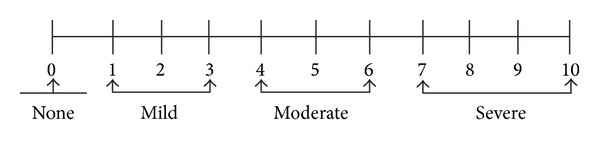
Severity of discomfort of acupuncture by VAS.

Discomfort of acupuncture refers to certain sensations felt by subjects during the treatment, such as aching, soreness, distension, heaviness, numbness, or dull pain that causes negative emotions, nervousness, fears, or hostility toward acupuncture. Discomfort of acupuncture was measured by the question: “How is your feeling of discomfort caused by acupuncture?” Subjects were asked to answer the question within 10 minutes after acupuncture treatment. The severity of discomfort was marked using visual analogue scale (VAS), a numerical scale from 0 to 10 where 0 means no discomfort and 10 means the severest discomfort (see Figure 1). Severity of discomfort was evaluated by values of VAS [7]. A value of 0 on the VAS indicates no discomfort, 1 to 3 indicates mild discomfort, 4 to 6 moderate discomfort, and 7 to 10 indicates severe discomfort.

#### 2.2.3. Acceptability of Acupuncture

Acceptability of acupuncture was assessed by the question: “How is your acceptability of acupuncture?” There were two options for selection, “Difficult to accept” or “Easy to accept.”

#### 2.2.4. Demographic Profile

To understand the influence of demographic characteristics toward discomfort and acceptability of acupuncture, the gender, age and education level were taken into consideration. Age was classified into seven segments: ≤19, 20–29, 30–39, 40–49, 50–59, 60–69, and ≥70. Educational level was determined based on the International Standard Classification of Education (ISCED) [8]. We classified educational level into three categories: primary education and below (ISCED level: 0-1), secondary education (ISCED level: 2–4), and tertiary education (ISCED level: 5–8).

#### 2.2.5. Acupuncture-Related Information

To explore acupuncture-related factors affecting discomfort and acceptability of acupuncture, five items were adopted in the questionnaire. These items included whether it was the first acupuncture experience, whether subjects were afraid of acupuncture needles, the extent of fear of pain, subjects' knowledge of acupuncture, and professional title of acupuncturist.

#### 2.2.6. Data Collection

The questionnaire was first given to 5 outpatients to assure clarity of concepts for subjects. The data was collected by two postgraduate students who majored in acupuncture and received formal training on questionnaire interviewing. To ensure research quality, a supervisor performed a spot check on completed questionnaires for completeness and consistency at the time of interview.

## 3. Statistical Analysis

Statistical analysis was performed using SPSS statistics 18.0. Binary logistic regression with backward stepwise was used to assess the relationship between acceptability of acupuncture and demographic, discomfort, or acupuncture-related variables. Chi-square test was used to test for association between acupuncture discomfort and demographic or acupuncture-related variables; variables that were shown to be significantly associated with discomfort of acupuncture were entered into multinomial logistic regression. *P* < 0.05 was considered statistically significant.

## 4. Results

### 4.1. Discomfort of Acupuncture

The average VAS value of 925 subjects' acupuncture discomfort was 2.66 ± 2.02, within the range of mild discomfort. 146 subjects (15.8%) did not feel any discomfort; the majority of subjects (53.9%) felt slight discomfort; 188 subjects (20.3%) felt moderate discomfort; and 92 (9.9%) felt severe discomfort. “Moderate discomfort” and “severe discomfort” responses were analyzed together (Table 1).

### 4.2. Acceptability of Acupuncture

Among 925 subjects, 750 subjects (81.1%) reported that acupuncture was easy to be accepted, and 175 (18.9%) reported that acupuncture was difficult to be accepted (Table 1).

### 4.3. Demographic Profile

Out of 925 subjects (mean age ± standard deviation: 47.97 ± 2.00 years), 40.2% were male and 59.8% were female. 33.7% had completed tertiary education, 58.6% secondary, and 7.7% primary education (Table 1). 

### 4.4. Acupuncture-Related Factors

There were 17.4% of subjects receiving acupuncture treatment for the first time, and 21.2% of subjects reported fear of needles. When asked how much they were afraid of pain, 32.9% of subjects chose “not at all”, 30.9% and 36.2% chose “very much” and “a little,” respectively.

Overall, most of the subjects were characterized by the following features: the previous acupuncture experience (82.6%), fear of acupuncture needles (78.8%), fear of pain (67.1%), little knowledge of acupuncture (80.8%), and seeking help from more qualified acupuncturists (82.9%). Details are shown in Table 1.

### 4.5. Factors Affecting Discomfort of Acupuncture

Results of chi-square tests showed that age (*χ* ^2^ = 32.83, *P* = 0.001), fear of needles (*χ* ^2^ = 34.15, *P* < 0.01), knowledge of acupuncture (*χ* ^2^ = 13.17, *P* = 0.01) and fear of pain (*χ* ^2^ = 53.75, *P* < 0.01), were significantly different among varying degrees of acupuncture discomfort.  Table 2 showed the multinomial logistic regression predicting the odds of reporting acupuncture discomfort as no discomfort or slight discomfort. 

Results showed that subjects reported more “moderate to severe discomfort” than “no discomfort” in several conditions: (1) if they had a fear of needles (OR = 0.26, 95% CI = 0.14–0.49); (2) if they were afraid of pain (a little versus not at all: OR = 0.22, 95% CI = 0.13–0.37; very much versus not at all: OR = 0.24, 95% CI = 0.14–0.41); (3) if they showed a better knowledge of acupuncture (a little versus not at all: OR = 0.43, 95% CI = 0.26–0.71, very well versus not at all: OR = 0.40, 95% CI = 0.21–0.77); and (4) if they were aged 20–29 (20–29 versus ≥70: OR = 0.35, 95% CI = 0.13–0.90).

In the comparisons between “slight discomfort” and “moderate to severe discomfort,” fear of needles, fear of pain and age rather than knowledge of acupuncture, showed significant differences. Subjects experienced more “moderate to severe discomfort” than “slight discomfort” if they were afraid of needles (OR = 0.50, 95% CI = 0.36–0.72) and pain (a little versus not at all: OR = 0.47, 95% CI = 0.32–0.69; very much versus not at all: OR = 0.50, 95% CI = 0.33–0.75), while subjects aged 40–49 reported more “slight discomfort” (40–49 versus ≥70: OR = 1.85, 95% CI = 1.04–3.30).

### 4.6. Factors Affecting Acceptability of Acupuncture

The influence of variables (i.e., discomfort of acupuncture, demographic, and acupuncture-related factors) on acceptability of acupuncture was analyzed by binary logistic regression with backward stepwise. During the analysis procedure, variables of gender, educational level, and age were removed from the equation successively. The residual variables were significantly associated with acceptability of acupuncture, including discomfort of acupuncture, first acupuncture experience, fear of needles, fear of pain, knowledge of acupuncture, and professional title of acupuncturist (Table 3).

Acupuncture discomfort and professional title of acupuncturist were positively associated with acupuncture acceptability. A lower discomfort (no discomfort versus moderate to severe discomfort: OR = 3.11, 95% CI = 1.45–6.72, *P* = 0.004; slight discomfort versus moderate to severe discomfort: OR = 1.90, 95% CI = 1.29–2.82, *P* = 0.001) and a higher professional title of acupuncturist (senior title versus primary title: OR = 3.22, 95% CI = 1.95–5.34, *P* < 0.001; middle title versus primary title: OR = 2.59, 95% CI = 1.58–4.23, *P* < 0.001) were significantly associated with greater willingness to accept acupuncture, while other variables were significantly associated with a decreased willingness to accept acupuncture, referring primarily to first acupuncture experience (OR = 0.62, 95% CI = 0.39–0.99, *P* = 0.047), a greater fear of pain (very much versus not at all: OR = 0.18, 95% CI = 0.10–0.31, *P* < 0.001; a little versus not at all: OR = 0.42, 95% CI = 0.24–0.74, *P* < 0.001), a better knowledge of acupuncture (very well versus not at all: OR = 0.32, 95% CI = 0.16–0.61, *P* = 0.001; a little versus not at all: OR = 0.32, 95% CI = 0.18–0.56, *P* < 0.001), and fear of needles (OR = 0.34, 95% CI = 0.22–0.51, *P* < 0.001).

## 5. Discussion

In present study, a female predominance was observed (female: male = 1.48 : 1), and the age distribution displayed a peak at around fifties. Our findings were consistent with previous report [9]. The educational levels of subjects in our study were mainly secondary education or higher. Majority of subjects believed that acupuncture induced slight or no discomfort (69.7%) and was easy to be accepted (81.1%). There were 11.4% of subjects considering that acupuncture was easy to be accepted, although they chose moderate or severe discomfort. This may be interpreted by subjects' strong expectation for good effectiveness of acupuncture. Overall, acupuncture causes a little discomfort and it can be accepted easily, which was consistent with the finding that 81% of subjects considered acupuncture process to be comfortable and relaxing [10].

Till now, there were few studies on acupuncture discomfort and acceptability. Fear of pain or needles, as negative emotional experience, could cause greater discomfort. Multinomial regression analysis showed that subjects experienced more discomfort if he or she had a stronger fear toward pain or needles. Compared with subjects aged ≥70, subjects aged 20–29 preferred to report “moderate to severe discomfort” while those aged 40–59 preferred to report “slight discomfort.” It was unexpected that a better knowledge of acupuncture led to more discomfort. In clinical practice, some patients showed great willingness to acupuncture treatment because of good effectiveness; however, their nervousness did not decrease with treatment sessions. Considering this fact, the result of knowledge of acupuncture may be somewhat understood. Nevertheless, further research is needed to understand the relationship between the knowledge and acupuncture discomfort. In addition, the previous acupuncture experience, educational level, and professional title of acupuncturist showed no significant influence on discomfort. so did the factor of gender. 

Acupuncture discomfort, which is a negative emotion like pain, can be predicted to be influenced by gender. The relationship between gender differences and pain has been reported a lot, but the conclusions are different. Most reports showed that males exhibited greater pain tolerance than females [11, 12], but a systematic review failed to show a clear and consistent pattern of gender differences in pain sensitivity [13]. Paradigm to investigate the role of gender in pain perception was mainly based on laboratory-induced thermal, pressure, chemical, or visceral pain, but the application of the paradigms for clinic pain is questionable [13]. In our study, we did not find a significant gender difference toward discomfort of acupuncture, which is similar to the result of systematic review. 

Binary logistic regression analysis of acupuncture acceptability showed that subjects could accept acupuncture more easily if they had less discomfort (no or slight discomfort) and were treated by acupuncturist with a higher professional title. However, they may have great difficulties in accepting acupuncture if they had a fear of pain or needles and a better knowledge of acupuncture. The more serious the negative experiences (e.g., discomfort, fear of pain and acupuncture) are, the more difficult the acupuncture is accepted by subjects. Patient without acupuncture experience showed more difficulty in accepting acupuncture treatment. However, the impact of the first acupuncture experience on acupuncture discomfort was little enough to be disregarded (*P* = 0.047). 

Subjects could consider acupuncture easy to be accepted when they were treated by an acupuncturist with a higher professional title, consistent with the previous report [14]. Our results showed that better knowledge of acupuncture may cause more discomfort, and lead to a decreased willingness to acupuncture acceptance. A possible reason was that subjects gained acupuncture knowledge mainly from their own experience, in which negative experience tended to be exaggerated. Further research is needed to understand the relationship of the knowledge, discomfort and acceptability of acupuncture. 

Overall, fear of needles and pain can cause more discomfort, resulting in decreased acceptance. However, it is worth mentioning that pain endurance can be influenced by pain-related self-efficacy and positive self-instruction [11]. Positive outcome expectancy also indicates a marked improvement in patients' self-reports of anxiety, pain and distress [15, 16]. Therefore, communication and encouragement before acupuncture can hopefully improve patients' fear of pain and acupuncture, thus reducing discomfort and increasing acceptance. Our results showed that a senior professional title of acupuncturist had barely any impact on discomfort, but it can help to increase acupuncture acceptance.

Due to limited staff and resources, our study was completed only in six acupuncture consulting rooms from three top TCM hospitals in Beijing; lower grade hospitals and communities were not included. Besides, purposive sampling instead of random sampling was used in the study. Therefore our study sample may not be sufficiently representative. Secondly, other factors that may affect acupuncture discomfort and acceptability were not included in this study, such as details of sociodemographic information (income, occupation, marital status, etc.), diagnosis of diseases, needling location selecting, and the needling depth. In addition, only the severities of the sensations induced by acupuncture were recorded; the type of sensation was neglected. Acupuncture sensations were categorized into two types, namely, sensations that cannot hurt as deqi, mainly including aching, soreness, and pressure, followed by tingling, numbness, dull pain, heaviness, warmth, fullness, and coolness, and sensations that can hurt as noxious stimulation, for example, sharp pain [17–19]. A certain type of sensation may contribute to both acupuncture discomfort and deqi. By neglecting the type of discomfort, we cannot differentiate useful discomfort constituting deqi from actual discomfort sensations. Finally, due to the cross sectional nature of this study, our findings should be interpreted as associations rather than implying causality.

## 6. Conclusions 

In conclusion, acupuncture is an acceptable therapy with less discomfort. Discomfort and acceptability of acupuncture can be affected by fear of pain, fear of needles, age, knowledge of acupuncture, and professional title of acupuncturist. Based on our results, methods to relieve discomfort and improve acceptance of acupuncture can be taken accordingly.

## Supplementary Material

This questionnaire is anonymous and used to collect data on subjects' discomfort of acupuncture, acceptability of acupuncture, demographic profile and acupuncture—related information.

## Figures and Tables

**Table 1:** Characteristics of subjects.

Variables	Number	%
How's your feeling of discomfort caused by acupuncture?		
No discomfort (VAS = 0)	146	15.8
Slight discomfort (1 ≤VAS ≤ 3)	499	53.9
Moderate to severe discomfort (4 ≤ VAS ≤ 10)	280	30.3
How is your acceptability of acupuncture?		
Difficult to accept	175	18.9
Easy to accept	750	81.1
Gender		
Female	553	59.8
Male	372	40.2
Age		
≤19	57	6.2
20–29	140	15.1
30–39	112	12.1
40–49	147	15.9
50–59	229	24.8
60–69	130	14.1
≥70	110	11.9
Educational level		
Primary education and below (ISCED level: 0-1)	71	7.7
Secondary education (ISCED level: 2–4)	542	58.6
Tertiary education (ISCED level: 5–8)	312	33.7
First acupuncture experience: Is this your first acupuncture experience?		
No	764	82.6
Yes	161	17.4
Fear of needles: Are you afraid of acupuncture needles?		
No	729	78.8
Yes	196	21.2
Fear of pain: How much are you afraid of pain?		
Not at all	304	32.9
A little	335	36.2
Very much	286	30.9
Knowledge of acupuncture: How much do you know about acupuncture?		
Not at all	217	23.5
A little	530	57.3
Very well	178	19.2
Professional title of acupuncturist		
Resident physician	158	17.1
Attending physician	363	39.2
Chief physician	404	43.7

**Table 2:** Multinomial logistic regression: Odds of subjects reporting less discomfort of acupuncture (*n* = 925).

Characteristic/subcategory	Discomfort of acupuncture: No. (%)			No discomfort^a^		Slight discomfort^a^	
	No	Slight	Moderate to severe	OR (95% CI)	*P* value	OR (95% CI)	*P* value
Age							
≥70	20 (18.1)	51 (46.4)	39 (35.5)	1.00		1.00	
≤19	7 (12.3)	35 (61.4)	15 (26.3)	1.12 (0.37–3.41)	0.841	2.02 (0.94–4.32)	0.070
20–29	8 (5.7)	76 (54.3)	56 (40.0)	0.35 (0.13–0.90)	0.030	1.16 (0.66–2.03)	0.605
30–39	11 (9.8)	66 (58.9)	35 (31.3)	0.78 (0.32–1.94)	0.596	1.57 (0.86–2.85)	0.142
40–49	22 (15.0)	88 (59.9)	37 (25.1)	1.37 (0.62–3.02)	0.443	1.85 (1.04–3.30)	0.037
50–59	52 (22.7)	114 (49.8)	63 (27.5)	1.69 (0.84–3.39)	0.138	1.39 (0.82–2.37)	0.226
60–69	26 (20.0)	69 (53.1)	35 (26.9)	1.21 (0.55–2.65)	0.638	1.31 (0.71–2.39)	0.379
Fear of needles: Are you afraid of acupuncture needles?							
No	132 (18.1)	407 (55.8)	190 (26.1)	1.00		1.00	
Yes	14 (7.2)	92 (46.9)	90 (45.9)	0.25 (0.14–0.48)	<0.001	0.50 (0.36–0.72)	<0.001
Fear of pain: How much are you afraid of pain?							
Not at all	79 (26.0)	171 (56.3)	54 (17.8)	1.00		1.00	
A little	36 (10.7)	176 (52.5)	123 (36.7)	0.22 (0.13–0.37)	<0.001	0.47 (0.32–0.69)	<0.001
Very much	31 (10.8)	152 (53.1)	103 (36.0)	0.24 (0.14–0.41)	<0.001	0.50 (0.33–0.75)	0.001
Knowledge of acupuncture: How much do you know about acupuncture?							
Not at all	51 (23.5)	110 (50.7)	56 (25.8)	1.00		1.00	
A little	72 (13.6)	291 (54.9)	167 (31.5)	0.43 (0.26–0.71)	0.001	0.85 (0.58–1.24)	0.394
Very well	23 (12.9)	98 (55.1)	57 (32.0)	0.40 (0.21–0.77)	0.006	0.82 (0.51–1.32)	0.423

OR: odds ratio; CI: confidence interval; ^a^The reference category is “moderate to severe discomfort”.

**Table 3:** Binary logistic regression: Odds of subjects reporting better acceptance of acupuncture (*n* = 925).

Characteristic/Subcategory	Acceptance of acupuncture		Odds Ratios	
	Difficult to accept	Easy to accept	OR (95% CI)	*P* value
First acupuncture experience: Is this your first acupuncture experience?				
No	135 (17.7)	629 (82.3)	1.00	
Yes	40 (24.8)	121 (75.2)	0.62 (0.39–0.99)	0.047
Fear of needles: Are you afraid of acupuncture needles?				
No	95 (13.0)	634 (87.0)	1.00	
Yes	80 (40.8)	116 (59.2)	0.34 (0.22–0.51)	<0.001
Fear of pain: How much are you afraid of pain?				
Not at all	21 (12.0)	283 (93.1)	1.00	1.00
A little	63 (18.8)	272 (81.2)	0.42 (0.24–0.74)	0.002
Very much	91 (31.8)	195 (68.2)	0.18 (0.10–0.31)	<0.001
Knowledge of acupuncture: How much do you know about acupuncture?				
Not at all	20 (9.2)	197 (90.8)	1.00	
A little	119 (22.5)	411 (77.5)	0.32 (0.18–0.56)	0.001
Very well	36 (20.2)	142 (79.8)	0.32 (0.16–0.61)	<0.001
Professional title of acupuncturist				
Resident physician	59 (37.3)	99 (62.7)	1.00	
Attending physician	63 (17.4)	300 (82.6)	2.59 (1.58–4.23)	<0.001
Chief physician	53 (13.1)	351 (86.9)	3.22 (1.95–5.34)	<0.001
Discomfort of acupuncture: How is your discomfort feeling caused by acupuncture?				
Moderate to severe discomfort	87 (31.1)	193 (68.9)	1.00	
Slight discomfort	79 (15.8)	420 (84.2)	1.90 (1.29–2.82)	0.001
No discomfort	9 (6.2)	137 (93.8)	3.11 (1.45–6.72)	0.004

OR: odds ratio; CI: confidence interval.
