# Supplementary material for: Prevalence and Correlates of Discomfort and Acceptability of Acupuncture among Outpatients in Chinese Acupuncture and Moxibustion Departments: A Cross-Sectional Study
Source: Evid Based Complement Alternat Med. 2013 Jun 24;2013:715480. doi: 10.1155/2013/715480 (PMC3707286; doi:10.1155/2013/715480)
Supplement: Supplementary file 1 — This questionnaire is anonymous and used to collect data on subjects' discomfort of acupuncture, acceptability of acupuncture, demographic profile and acupuncture—related information. [file 715480.f1.pdf]

Discomfort and Acceptability of Acupuncture Questionnaire

Initial Number: |__|__|__| Date: |__|__| |__|__| 201|__|
Day Month Year

Acupuncture shows an increasingly wide utilization worldwide. We are trying to find out severity of discomfort it induces, its acceptability and related factors. We would be grateful if you could answer the following questions, thinking about your latest acupuncture experience. Tick one box for question 2-8, 10.

**1. please write in your date of birth:**	|__|__| |__|__| |__|__|__|__| Day Month Year
**2. Are you:**	Female ☐ Male ☐
**3. Your educational level:**	Primary education and below ☐<br>Secondary education ☐<br>Tertiary education ☐
**4. Is this your first acupuncture experience?**	Yes ☐<br>No ☐
**5. Are you afraid of acupuncture needles?**	Yes ☐<br>No ☐
**6. How much are you afraid of pain?**	Very much ☐<br>A little ☐<br>Not at all ☐
**7. How much do you know of acupuncture?**	Not at all ☐<br>A little ☐<br>Very well ☐
**8. Professional title of your acupuncturist:**	Resident physician ☐<br>Attending physician ☐<br>Chief physician ☐

9. How is your feeling of discomfort caused by acupuncture?

Please ring a number between 0 (none) and 10 (most severe).

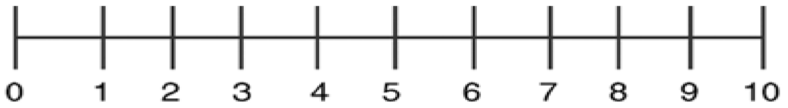

none Severest

10. How is your acceptability of acupuncture?

Difficult to accept ☐

Easy to accept ☐

Thank you very much for answering these questions.
